# Interpretable Machine Learning for Cognitive Aging: Handling Missing Data and Uncovering Social Determinants

**DOI:** 10.1093/geroni/igaf122.4146

**Published:** 2025-12-31

**Authors:** Xi Mao, Hairong Wang, Lingchao Mao, Jingyu Li, Zhendong Wang, Xuelei Ni

**Affiliations:** University of Texas Rio Grande Valley, Brownsville, Texas, United States; The University of Texas at Austin, Austin, Texas, United States; Georgia Institute of Technology, Atlanta, Georgia, United States; Georgia Institute of Technology, Atlanta, Georgia, United States; Shandong Mental Health Center, Jinan, Shandong, China (People’s Republic); Kennesaw State University, Kennesaw, Georgia, United States

## Abstract

Early detection of Alzheimer’s disease (AD) is critical, as neuropathologic change and modifiable social behavioral risks accumulate years before diagnosis. Identifying higher-risk individuals earlier enables prevention, timely care, and more equitable resource allocation. We study prediction of cognitive performance from social determinants of health (SDOH) using the NIH NIA supported PREPARE Challenge Phase 2 dataset, derived from the nationally representative Mex-Cog cohort within the 2003 and 2012 Mexican Health and Aging Study (MHAS). The target is a validated composite cognitive score covering orientation, immediate and delayed memory, attention, language, constructional praxis, and executive function, which derived from 2021 and 2016 MHAS. We curated features across demographic, socioeconomic, health, lifestyle, psychosocial, and healthcare access domains to capture multidimensional social and behavioral influences on cognitive aging. Substantial missingness was addressed with a singular value decomposition (SVD)-based imputation pipeline, treating continuous and categorical variables separately. This leverages latent feature correlations to recover missing values while balancing reliability and scalability. To better understand the relationship between input features and the composite cognitive score, we conducted a thorough post hoc analysis of the top contributing features, examining the mechanism of these features are associated with cognitive scores. The study further stratified the analysis by age group to explore whether the most predictive features differ across life stages. The proposed framework demonstrates robustness, interpretability, and computational efficiency, underscoring its potential as a practical modeling strategy for MHAS data in AD and MCI detection with substantial missingness across both continuous and categorical features.

